# Ensuring Reproducibility and Deploying Models with the Image2Radiomics Framework: An Evaluation of Image Processing on PanNET Model Performance

**DOI:** 10.3390/cancers17152552

**Published:** 2025-08-01

**Authors:** Florent Tixier, Felipe Lopez-Ramirez, Emir A. Syailendra, Alejandra Blanco, Ammar A. Javed, Linda C. Chu, Satomi Kawamoto, Elliot K. Fishman

**Affiliations:** 1 The Russell H. Morgan Department of Radiology and Radiological Science, School of Medicine, Johns Hopkins University, Baltimore, MD 21205, USA; jlopezr5@jh.edu (F.L.-R.); msyaile1@jhu.edu (E.A.S.); ablanco5@jhmi.edu (A.B.); lchu1@jhmi.edu (L.C.C.); skawamo1@jhmi.edu (S.K.); efishman@jhmi.edu (E.K.F.); 2 Department of Surgery, The NYU Grossman School of Medicine and NYU Langone Health, New York, NY 10016, USA; ammar.javed@nyulangone.org

**Keywords:** radiomics, image processing, machine learning, model validation, reproducibility, Image2Radiomics

## Abstract

Background/Objectives: To evaluate the importance of image processing in a previously validated model for detecting pancreatic neuroendocrine tumors (PanNETs) and to introduce Image2Radiomics, a new framework that ensures reproducibility of the image processing pipeline and facilitates the deployment of radiomics models. Methods: A previously validated model for identifying PanNETs from CT images served as the reference. Radiomics features were re-extracted using Image2Radiomics and compared to those from the original model using performance metrics. The impact of nine alterations to the image processing pipeline was evaluated. Prediction discrepancies were quantified using the mean ± SD of absolute differences in PanNET probability and the percentage of classification disagreement. Results: The reference model was successfully replicated with Image2Radiomics, achieving a Cohen’s kappa coefficient of 1. Alterations to the image processing pipeline led to reductions in model performance, with AUC dropping from 0.87 to 0.71 when image windowing was removed. Prediction disagreements were observed in up to 45% of patients. Even minor changes, such as switching the library used for spatial resampling, resulted in up to 21% disagreement. Conclusions: Reproducing image processing pipelines remains challenging and limits the clinical deployment of radiomics models. While this study is limited to one model and imaging modality, the findings underscore a common risk in radiomics reproducibility. The Image2Radiomics framework addresses this issue by allowing researchers to define and share complete processing pipelines in a standardized way, improving reproducibility and facilitating model deployment in clinical and multicenter settings.

## Simple Summary

This study investigates how image processing choices can affect the performance and reliability of radiomics models, using a previously validated model for detecting pancreatic neuroendocrine tumors from CT scans. We found that even small changes in preprocessing can significantly alter predictions, with classification discrepancies in up to 45% of cases. To address this issue, we developed Image2Radiomics, a framework that ensures reproducibility of image processing and simplifies model deployment. While this study focuses on a single model, the results highlight a common risk in radiomics reproducibility. Image2Radiomics helps bridge the gap to clinical practice by making these steps consistent and transparent.

## 1. Introduction

Since radiomics was defined in 2012 as the high-throughput extraction of features from medical images [1], evidence supporting the predictive capabilities of such features to predict patient outcomes, response to therapy, and capture underlying biological pathways has been demonstrated numerous times. This evidence extends across diverse image modalities, including CT, PET, and MRI [2,3,4,5], and encompasses a range of pathologies within fields such as oncology [6,7], neurology [8], and cardiology [9].

Recent studies have investigated solutions to harmonize images and radiomics features in order to reduce the effect on extracted data at different centers on the features and allow them to build multicentric radiomics models. This harmonization can be performed on the image space by using algorithms such as histogram matching, or more complex solutions based on convolutional neural networks [10,11]. Harmonization in the image space helps to make radiomics features comparable across patients from different batches (center, scanner manufacturer or image acquisition and reconstruction protocols). However, this harmonization is not perfect, and statistical differences can be found in radiomics extracted from different configurations even after image harmonization [10]. For this reason, often a features harmonization is performed in addition to image harmonization. The most commonly method used is called ComBat [12,13], which allows for transforming the data to make them match data of a reference center [13]. The combination of image and feature harmonization significantly improved the performance of radiomics in multicentric datasets [14].

In addition, important improvements in the methodology of radiomics analysis help to ensure that radiomics features are extracted in the same way across studies by using the image standardization initiative guidelines (IBSI) [15,16]. The IBSI also provides publicly available datasets to ensure comparable results across different radiomics software and libraries and facilitate the development and validation of new in-house solutions.

However, radiomics still faces challenges in transitioning from research studies to practical clinical applications due to two major constraints [17,18,19]. First, radiomics models often rely on features that were post-processed using statistics (e.g., mean or standard deviation) and covariates (e.g., scanner manufacturer or reconstruction parameters), which are used to standardize the features (Z-score normalization, ComBat harmonization), before building the models. These details, necessary to apply the models to new patients, are rarely shared. Second, even with a detailed description, it can be difficult to fully reproduce the image processing pipeline prior to radiomics feature extraction, and the impact of changes in the pipeline on the classification results of radiomics models has not been fully evaluated.

In this study we introduce the Image2Radiomics framework designed to ensure reproducibility of image processing pipelines and facilitate the deployment of radiomics models. As a secondary objective, we aim to replicate a previously validated model for distinguishing patients with pancreatic neuroendocrine tumors (PanNETs) from control patients based on radiomics features extracted from CT images [20] and evaluate how alterations in the image processing pipeline can impact the classification results.

## 2. Materials and Methods

### 2.1. Image2Radiomics Framework

**Figure 1:**
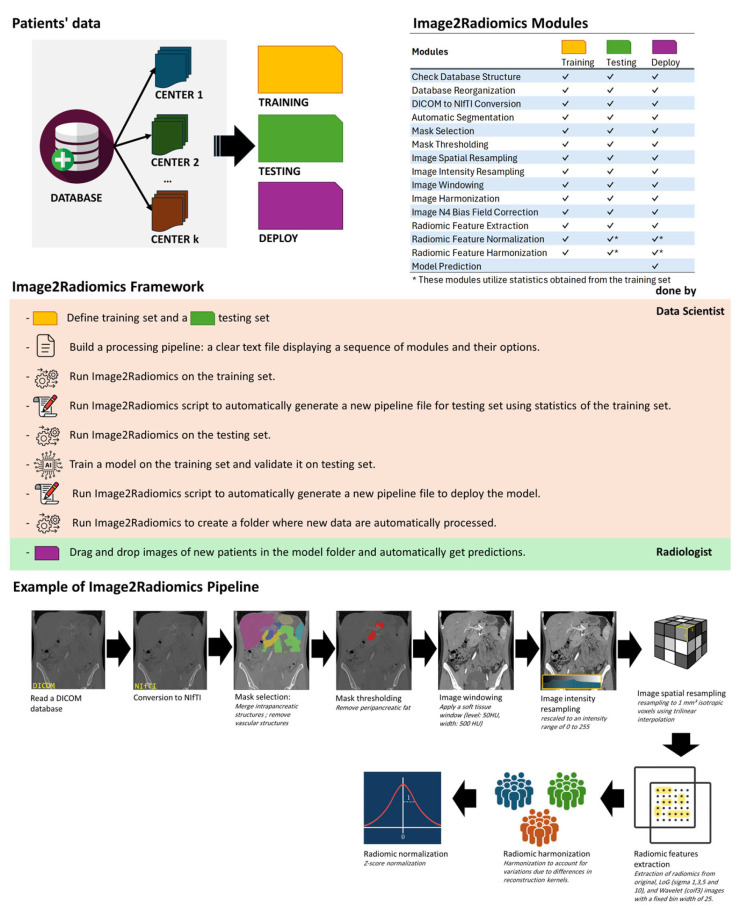
Overview of the Image2Radiomics (I2R) framework. Before using the framework, image data from one or multiple centers must be split into training and testing sets. Later, I2R can be used to deploy the model on new data. The process begins by defining a radiomics pipeline using the modules listed in the upper-right panel. I2R is then run on the training set using this pipeline. A dedicated script modifies the pipeline for the testing set, ensuring that modules relying on training-derived statistics (e.g., normalization or harmonization) are correctly applied. At this stage, I2R provides radiomics features for both training and testing sets, which can be used to train and validate a model. A final script enables deployment by generating a version of the pipeline that automatically processes new patient data and outputs model predictions. The full set of pipelines, preprocessing statistics, batch harmonization information, and trained model can then be shared for reuse across centers. The bottom section of the figure illustrates an example of a processing pipeline that can be automated with the I2R framework.

We developed a comprehensive framework (Image2Radiomics) designed for the extraction of radiomics features, with the added capability to apply consistent preprocessing to the data before or after feature extraction. The framework performs data processing through distinct modules, each responsible for a specific task (Figure 1). These tasks are executed in a predefined sequence by using a pipeline (structured text file) that specifies the order of modules and their respective configuration parameters. The pipeline file and the radiomics statistics file generated by Image2Radiomics can then be shared to allow external centers to apply the model following the exact same processing pipeline with new data. The Image2Radiomics framework is implemented in Python and has been tested with Python 3.11 on Linux. The framework relies on several widely used packages and programs for image processing and radiomics analysis, including SimpleITK [21], dicom2nifti, NiBabel, C3D (itk-snap) [22], RT-Utils [23], neuroCombat [12], PyRadiomics [24], and TotalSegmentator [25]. Image2Radiomics consolidates all these tools into a single framework, offering an end-to-end solution. The framework supports parallel processing, making it suitable for use on both everyday computers and high-performance computing (HPC) clusters. It is compatible with the Simple Linux Utility for Resource Management (SLURM) and Sun Grid Engine (SGE) job scheduling systems. Image2Radiomics can be downloaded on GitHub at https://github.com/FlTixier/I2R (accessed on 29 July 2025).

### 2.2. PanNETsModel

**Figure 2:**
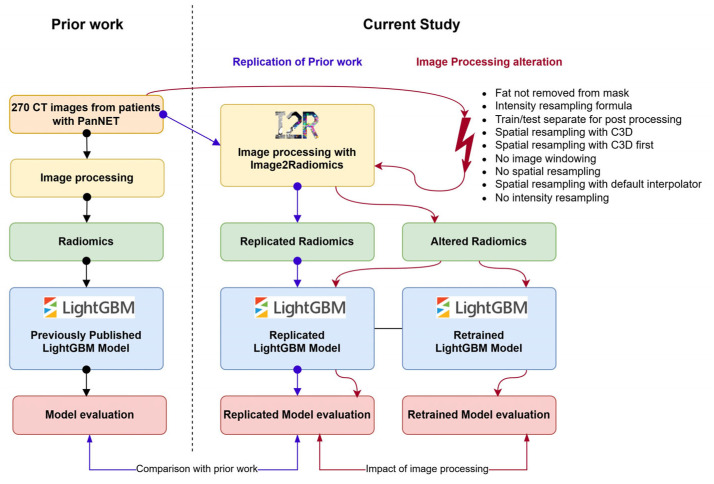
Flowchart of LightGBM Model Replication and Image Processing Alterations.

We tested a previously developed classifier model based on the LightGBM algorithm [26], utilizing 10 radiomics features selected from the contrast-enhanced CT in arterial phase [20]. This model operates on radiomics features derived from a segmentation mask that merges intrapancreatic structures, including pancreatic tissue, main pancreatic duct, cysts, and tumors, while excluding vascular structures, the common bile duct, the duodenum and the spleen. Segmentation masks were edited to remove peripancreatic fat by eliminating voxels with Hounsfield units (HU) less than zero [27]. CT volumes were harmonized using a soft tissue window (level: 50 HU, width: 500 HU) and rescaled to an intensity range of 0 to 255. The CT images were then resampled to 1 mm^3^ isotropic voxels using trilinear interpolation with PyRadiomics. Radiomics features were extracted with a bin width of 25. Following extraction, the features underwent ComBat harmonization [12,13] to account for kernel variability, using iterative kernel as a reference, and were subsequently normalized using z-score normalization (see pipeline example in Figure 1). The classifier was trained on 176 patients and hyperparameters were tuned using a 5-fold cross validation on the training set. Then the model performance was validated on a testing set of 94 patients. A full description of the model is available in the original paper [20]. The image processing pipeline used in the original paper was developed without Image2Radiomics. In this study the Image2Radiomics framework was used to replicate the radiomics features extraction for the same patients and following the processing pipeline (Figure 2). Modeling was performed on arterial phase images using the same patients in the training and testing sets as defined in the study by Lopez-Ramirez et al. [20], ensuring consistency with the original methodology. This model was chosen because the image processing pipeline involves a large portion of the modules in Image2Radiomics, with radiomics features and classification results available for each individual patient.

### 2.3. Image Processing Pipeline Alteration

To evaluate the impact of image processing, we modified the pipeline with alterations affecting segmented volume, intensity resampling, spatial resampling or radiomics post-processing (Supplementary Table S2). These modifications included adjustments to key pipeline steps, as well as subtle changes in how specific steps were implemented. We considered the following configurations: (1) removing peripancreatic fat in the segmentation mask, (2) the intensity resampling was performed using NumPy library binning functions, (3) training and testing sets were processed separately in radiomics post-processing, (4) the spatial resampling was performed with C3D [22], (5) spatial resampling was performed with C3D at the beginning of the image processing pipeline, (6) image windowing was removed from the pipeline, (7) spatial resampling was removed from the pipeline, (8) the spatial resampling was performed with the default interpolator in pyradiomics (B-Spline) [24], and (9) the intensity resampling was removed from the pipeline.

The radiomics extracted from these 9 pipeline variations were used to evaluate the classification accuracy in the testing set using the original LightGBM model. Additionally, we also used the radiomics extracted from pipeline variations to retrain different models using the same hyperparameters and evaluated the classification performance on the testing set. The first configuration represents a scenario where someone is attempting to use an existing model, while the second configuration represents an effort to replicate results. In the following, we refer to these two configurations as the non-retrained and retrained models, respectively.

### 2.4. Model Evaluation

To compare the performance of the models with different configurations, we used the area under the receiver operating characteristic curve (AUC-ROC). For each patient, the LightGBM model provides a probability between 0 and 1 for the presence of a PanNET in the pancreas. We applied a threshold of 0.5 to convert this probability into a binary classification task of either control or PanNET, and computed the following metrics: accuracy, sensitivity, specificity, positive predictive value, and F1-score. We quantified the mean ± standard deviation (SD) of the absolute probability difference between classifications from two different image processing pipelines, using both the retrained and non-retrained configurations, across all patients, control patients, and PanNET patients in the testing set. We also looked at the percentage of discordance from the original model in the classification from different image processing pipelines and calculated Cohen’s kappa coefficient to measure agreement with the original model.

## 3. Results

### 3.1. Replication of PanNET Study

Radiomics features extracted with Image2Radiomics were identical to the one used in the original PanNET study [20]. We also found the same patients classification prediction (0% of classification discordance on the testing set, Cohen’s Kappa: 1) and the exact same probability output from the LightGBM model.

### 3.2. Impact of the Altered Image Processing Pipelines on the Model Performance

**Figure 3:**
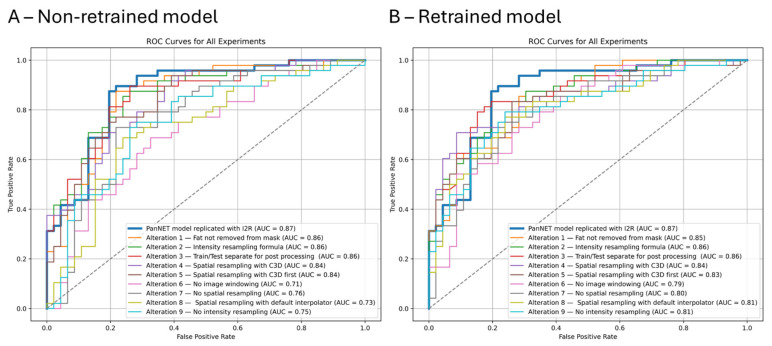
Receiver operating curves for (**A**) Non-retrained models and (**B**) retrained models.

When the PanNET classification model was not retrained, altering the image processing pipeline resulted in AUC-ROCs ranging from 0.71 to 0.86, compared to 0.87 in the original model, respectively (Figure 3A and Supplementary Table S1). The alterations that had the least impact on the model’s performance included removing peripancreatic fat from the mask (AUC-ROC: 0.86), changing the formula for intensity resampling (AUC-ROC:0.86), applying radiomics feature standardization separately on the training and testing sets (AUC-ROC:0.86), and omitting intensity resampling (AUC-ROC:0.85). On the other hand, the changes that most negatively affected model performance were removing image windowing (AUC-ROC:0.71), removing spatial resampling (AUC-ROC:0.76), or performing spatial resampling with the default interpolator (AUC-ROC: 0.73). Removing spatial resampling resulted in the model with the lowest specificity (0.37), while removing image windowing resulted in the model with the lowest sensitivity (0.52) (Supplementary Table S1).

When evaluating retrained models, altering the image processing pipeline resulted in AUC-ROCs ranging from 0.79 to 0.86, compared to 0.87 in the original model, respectively (Figure 3B and Supplementary Table S1). Similarly, omitting image windowing or spatial resampling had the most negative impact on the models, resulting in sensitivities of 0.79 in both and specificities of 0.61 and 0.67, respectively (Supplementary Table S1).

### 3.3. Impact of the Altered Image Processing Pipelines on the Model Predictions

**Figure 4:**
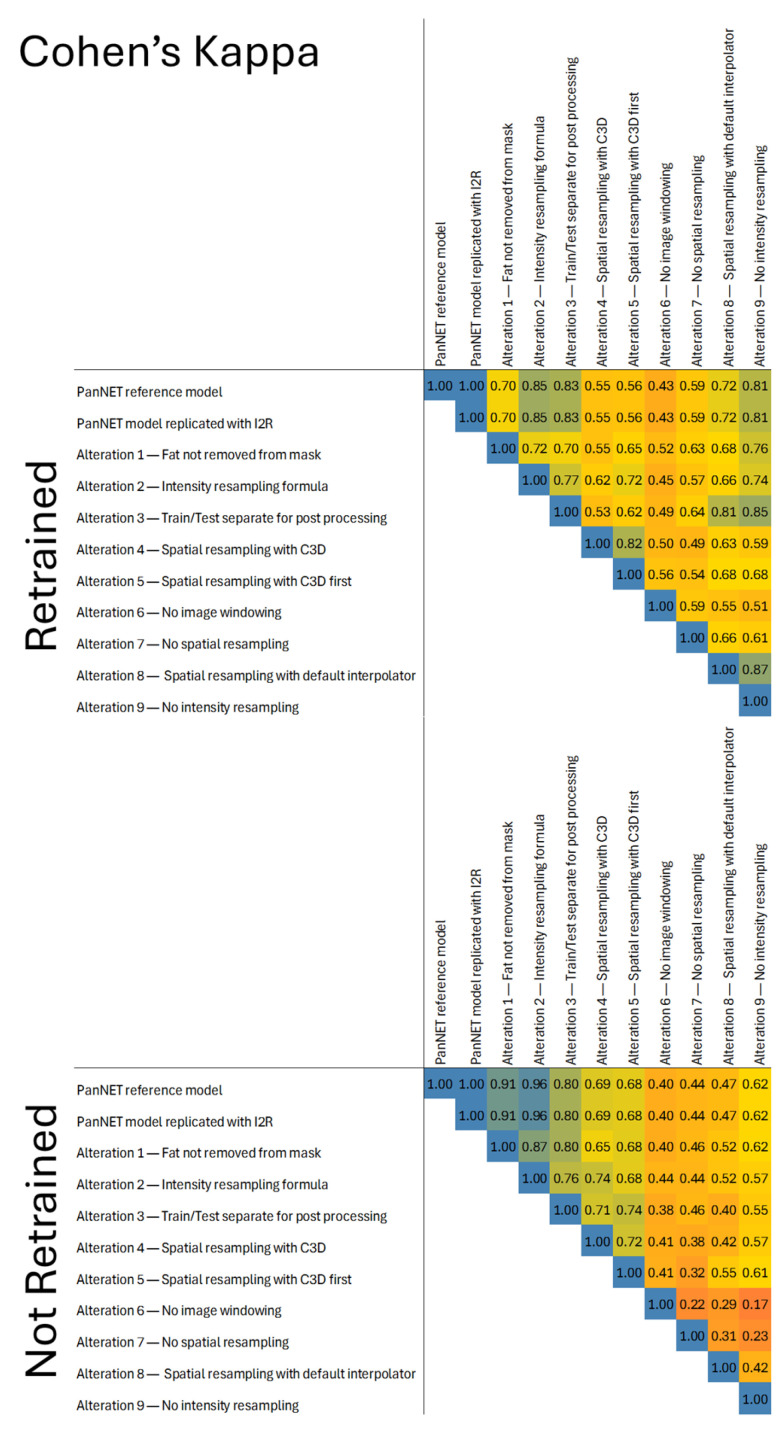
Heatmap of Cohen’s Kappa values comparing agreement across different pipeline configurations for retrained models (**top**) and non-retrained models (**bottom**). Rows and columns represent specific alterations to the image processing pipeline.

**Figure 5:**
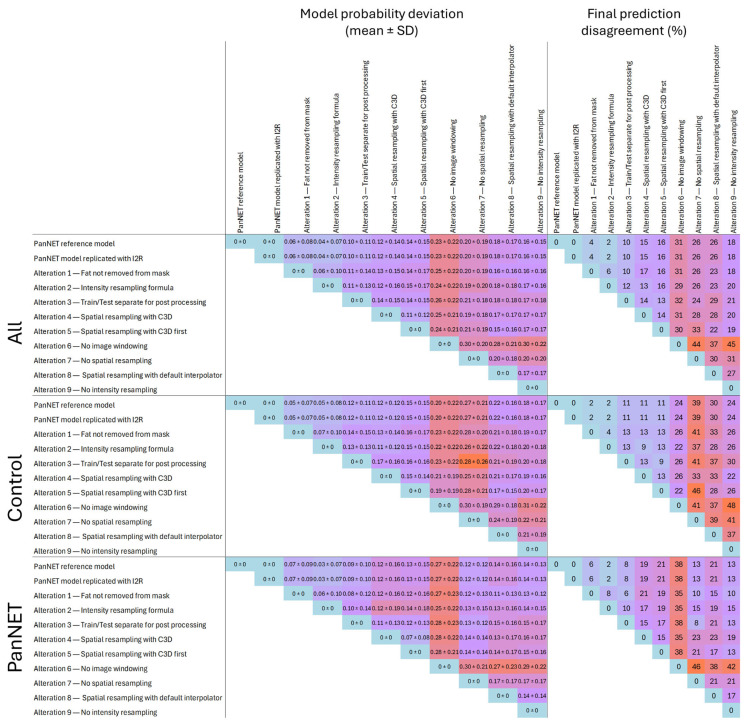
**Left**: Mean ± standard deviation of the absolute probability difference between the reference model (not retrained) applied to patients with radiomics features extracted using different preprocessing configurations and the original reference model across all patients, ‘normal’ patients, and PNET patients in the testing set. **Right**: Percentage of disagreement between the final predictions of the reference model applied to patients with radiomics features extracted using different preprocessing configurations and the original reference model across all patients, ‘normal’ patients, and PNET patients in the testing set.

Without retraining the model, we found a mean variation in the PanNET probability of up to 0.30 ± 0.22, when comparing predictions without image windowing and without intensity resampling on the testing set. Using a threshold of 0.5 to classify patients as PanNET or control, we observed up to 45% disagreement with the original model in the final classification (Cohen’s Kappa 0.17). Image windowing and spatial resampling were identified as the two steps with the most impact on the final probability. Conversely, not removing fat from the mask or using an alternative intensity resampling formula resulted in smaller changes in the probability, with mean ± SD differences of 0.06 ± 0.08 and 0.04 ± 0.07, respectively, compared to the predictions from the reference model. These changes resulted in final classification disagreements of 4% and 2%, respectively (Cohen’s Kappa: 0.91 and 0.96, respectively). When examining the probability differences for PanNET and control patients separately, omitting image windowing caused larger probability differences in the PanNET group than in the control group. In contrast, alterations involving spatial or intensity resampling resulted in smaller probability differences in the PanNET group (Figure 4 and Figure 5).

**Figure 6:**
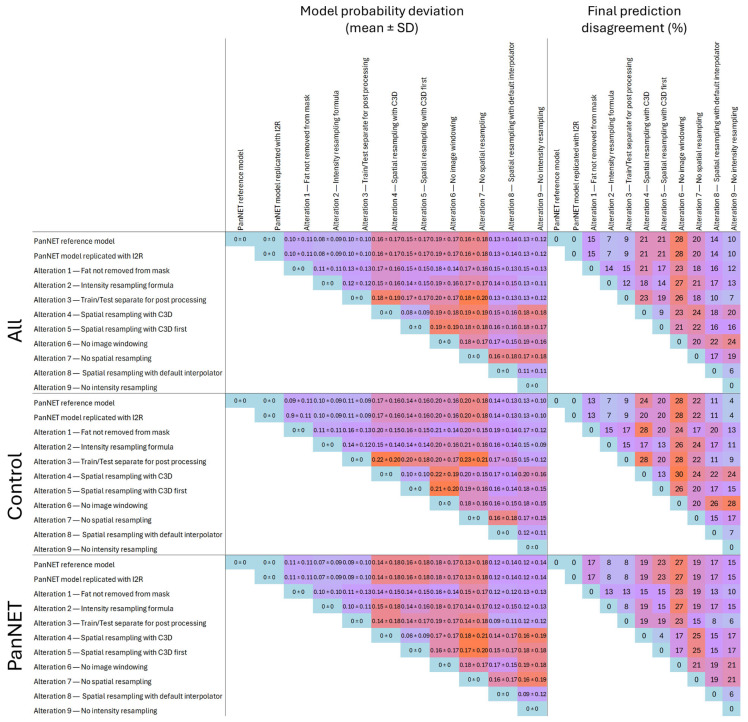
**Left**: Mean ± standard deviation of the absolute probability difference between the retrained models using different configurations and the reference model across all patients, ‘normal’ patients, and PNET patients in the testing set. **Right**: Percentage of disagreement between the final predictions of the retrained models and the reference model, across all patients, ‘normal’ patients, and PNET patients in the testing set.

With the model retrained on radiomics from altered pipelines, the largest mean ± SD probability differences were observed for pipelines without spatial resampling and pipelines without image windowing, with mean differences ranging between 0.18 ± 0.14 and 0.20 ± 0.17. For the final classification, the highest discordance of 28% was found when comparing the reference model with the model without image windowing. The smallest differences were found for models using radiomics from pipelines without fat removal from the mask, pipelines with an alternative intensity resampling formula, and pipelines with radiomics post-processed separately for the train and testing sets, with mean differences in the probability ranging between 0.08 ± 0.09 and 0.13 ± 0.13. For the final classification, a minimum discordance of 6% was observed when comparing the model built from radiomics without intensity resampling to the model using spatial resampling with the default interpolator (Cohen’s kappa: 0.87). Models using an alternative resampling formula and those with radiomics post-processed separately on the train and test sets also showed low classification discordance, at 7% and 9%, respectively (Cohen’s kappa: 0.85 and 0.83, respectively) (Figure 5). When examining the differences for PanNET and control patients separately, we found some configurations that resulted in higher discordance in the control group than in the PanNET group. For example, the comparison between the model trained with resampling using C3D and the model without image windowing led to 30% misclassification in the control group and 17% in the PanNET group. Conversely, the comparison between the model with no intensity resampling and the reference model resulted in 4% misclassification in the control group and 15% in the PanNET group. However, we did not find any consistent imbalance in the discordance percentages between control and PanNET groups for specific altered pipelines (Figure 4 and Figure 6).

## 4. Discussion

In this paper, we studied the impact of image processing pipelines on radiomics model classification and introduced a new radiomics framework, Image2Radiomics, to fully reproduce the pipelines used to build prediction models.

We found that removing image windowing or spatial resampling from the radiomics pipeline of the PanNET model had the most significant impact on the classification results. While it is unlikely that such essential steps would be omitted when replicating a radiomics study or applying a model to new patients, this finding highlights their critical importance in radiomics modeling. Consequently, for clinical reproducibility, it is fundamental to ensure that both image windowing and spatial resampling are applied consistently to avoid significant mismatches. Moreover, we found that other steps seemingly trivial, such as performing supposably the same spatial resampling with different libraries (C3D [22] or PyRadiomics [24]), resulted in classification disagreements for 21% of patients in the retrained model. These two resampling tools are written in C++ and Python, and use ITK and SimpleITK libraries, respectively. The exact cause of the observed classification differences requires further investigation. Similarly, applying spatial resampling at the beginning or end of the pipeline led to classification disagreements in up to 14% of patients, and using the default interpolator (B-spline) in PyRadiomics versus trilinear resampling resulted in classification disagreements in up to 26% of patients. These classification disagreements could have important consequences for patients [28]. Depending on the intended use of the model, such discrepancies could lead to incorrect diagnoses, potentially resulting in unnecessary follow-up imaging, invasive procedures such as biopsies or surgery, or, conversely, missed opportunities for early treatment initiation. To accurately reproduce the steps in the image processing pipeline, a precise description is essential.

Other authors have investigated the impact of image preprocessing on radiomics features [2,15,29,30], but to the best of our knowledge, this is the first attempt to examine its impact on predictions from radiomics models and the potential limitations in deploying existing models due to errors in fully replicating the image pre-processing. The IBSI guidelines emphasized the importance of image processing and provided recommendations to ensure consistent results after evaluating multiple processing configurations [15]. This paper marked a significant step forward for reproducible radiomics studies. Wichtmann et al. found that image processing has a significant impact on the values of extracted radiomics features, and through a test–retest experiment, they identified that achieving the best repeatability depends not only on image processing but also on the MRI sequences used [30]. Similarly, Janna E. van Timmeren et al. described that test–retest analyses should be performed for each radiomics study to account for factors such as scanners, imaging protocols, reconstruction methods, and time points, which directly impact the repeatability of results [29]. Therefore, image processing in radiomics studies should be selected based on the specific needs of each study but must aim to be easily reproducible. The Image2Radiomics framework is based on the PyRadiomics library for the radiomics module, which complies with IBSI guidelines [15,16]. Additional modules can also be executed before or after radiomics extraction to cover all aspects of the image processing pipeline (Figure 1). These steps can be highly specific to the study (e.g., image modality, disease, organ of interest) [2] and Image2Radiomics helps to explore various processing configuration, ensures the reproducibility of the results and facilitates the deployment of radiomics models for application to new patients. In addition, studies involving radiomics should include a complete and transparent description of the image processing pipeline, detailing each step, all parameters and options used, the order in which the steps are applied, and the software libraries (including version numbers) employed for each step [31].

AI model deployment is already facilitated by other tools such as MONAI [32] and initiatives like those led by QIBA [33], which aim to support the translation of research tools into clinical practice. Image2Radiomics shares similar goals but distinguishes itself by being specifically designed for radiomics models, in contrast to general-purpose frameworks like MONAI, which are primarily optimized for deep learning. Moreover, Image2Radiomics enables model deployment and reuse without requiring users to write code, thereby lowering the technical barrier for clinical researchers.

In this study, disagreement results from non-retrained models reflect the outcomes we can expect when applying an existing model to new patients, while disagreement results from retrained models represent the outcomes we can expect when attempting to reproduce a model by retraining it. The retrained models used the same hyperparameters as the original model, as the goal was to assess the impact of the image processing when attempting to reproduce an existing model. Optimizing the hyperparameters for the altered radiomics pipeline could potentially have led to better classification results than the original model, but this was beyond the scope of this study.

It is important to note that, in most cases, applying an existing model to new data or retraining it can be impractical and Tugba Akinci D’Antonoli et al. reported that only 3% of radiomics papers shared both code and data [34]. In addition, if the model does not use radiomics standardization, it is unlikely to perform well with data from different centers, scanners, reconstruction methods, or acquisition protocols [35,36]. When standardization is applied, statistics on the radiomics data, batch information, and the extracted radiomics features used to build the model are required to ensure consistent standardization for new patients, but this information is rarely shared with the model. The Image2Radiomics framework provides an efficient way to share radiomics models across centers, ensuring that the necessary information for standardization and reproducibility is available and can therefore facilitate the deployment of radiomics models.

This study has some limitations, as we only investigated the variations on a model based on a gradient boosting machine (LightGBM), which may be more sensitive to small variations and dependent on hyperparameters than other machine learning models. Here, the standardized preprocessing provided by Image2Radiomics is designed to improve model stability, in the same way that the nnU-Net framework has improved the reliability of U-Net-based models. However, more robust machine learning architectures may lead to fewer misclassifications and should be explored in future studies [37]. Additionally, we focused on the impact of the processing pipeline on the classification results rather than on the radiomics features themselves. This choice was made because the primary goal was to evaluate the reliability of classification results based on adherence to the processing pipeline. However, further investigation into the radiomics features is necessary to fully understand the impact of the image processing pipeline. Different results may have been obtained if the model had relied on features with different sensitivity to preprocessing changes. We limited the study to this single use case because publicly available radiomics models, even those based on public datasets, often do not provide all the necessary files to fully replicate the study. However, we anticipate that our results are generalizable, and the Image2Radiomics framework will facilitate extending the results presented in this study to other tumor sites and clinical outcomes.

## 5. Conclusions

In conclusion, we found that even small changes to the image processing pipeline can significantly alter radiomics model predictions, with classification discrepancies observed in up to 45% of patients. This highlights the need for reproducible preprocessing to ensure reliable clinical application. The Image2Radiomics framework introduced in this study allows researchers to fully define, share, and automate processing pipelines, ensuring reproducibility and simplifying model deployment. While this study is limited to a single model and imaging modality, it demonstrates the potential consequences of inconsistent preprocessing. Future work should assess generalizability across diseases, modalities, and model architectures. Such frameworks are necessary, along with standards for documenting and sharing radiomics pipelines, to make radiomics models trustworthy and clinically usable.

## Data Availability

The datasets analyzed in this study are not publicly available due to privacy or ethical restrictions.

## Supplementary Materials

The following supporting information can be downloaded at: https://www.mdpi.com/article/10.3390/cancers17152552/s1, Table S1. Model performance with or without retraining for the reference model and model applied on data obtained from altered pipelines. Diagnostic metrics were obtained with a probability cut-off of 0.5 between the categories. Table S2. Overview of the nine altered image processing pipeline configurations evaluated.

## Abbreviations

The following abbreviations are used in this manuscript:

AUC	Area Under the Curve
CT	Computed Tomography
HPC	High-Performance Computing
HU	Hounsfield Unit
IBSI	Image Biomarker Standardisation Initiative
LightGBM	Light Gradient Boosting Machine
MRI	Magnetic Resonance Imaging
PanNET	Pancreatic Neuroendocrine Tumor
PET	Positron Emission Tomography
SD	Standard Deviation
SLURM	Simple Linux Utility for Resource Management
SGE	Sun Grid Engine
