# Supplementary material for: Ensuring Reproducibility and Deploying Models with the Image2Radiomics Framework: An Evaluation of Image Processing on PanNET Model Performance
Source: Cancers (Basel). 2025 Aug 1;17(15):2552. doi: 10.3390/cancers17152552 (PMC12346810; doi:10.3390/cancers17152552)
Supplement: Supplementary file 1 [file cancers-17-02552-s001.zip › cancers-3750799-supplementary.pdf]

Suppl. Table S1. Model performance with or without retraining for the reference model and model applied on data obtained from altered pipelines. Diagnostic metrics were obtained with a probability cut-off of 0.5 between the categories.

		AUC-ROC	Accuracy	Sensitivity	Specificity	PPV	F1-Score
Non-retrained model	PanNET reference model	0.87	0.83	0.90	0.76	0.80	0.84
	PanNET model replicated with I2R	0.87	0.83	0.90	0.76	0.80	0.84
	Alteration 1 — Fat not removed from mask	0.86	0.83	0.88	0.78	0.81	0.84
	Alteration 2 — Intensity resampling formula	0.86	0.81	0.88	0.74	0.78	0.82
	Alteration 3 — Train/Test separate for post processing	0.86	0.82	0.90	0.74	0.78	0.83
	Alteration 4 — Spatial resampling with C3D	0.84	0.74	0.83	0.65	0.71	0.77
	Alteration 5 — Spatial resampling with C3D first	0.84	0.76	0.77	0.74	0.76	0.76
	Alteration 6 — No image windowing	0.71	0.63	0.52	0.74	0.68	0.59
	Alteration 7 — No spatial resampling	0.76	0.66	0.94	0.37	0.61	0.74
	Alteration 8 — Spatial resampling with default interpolator	0.73	0.66	0.77	0.54	0.64	0.70
	Alteration 9 — No intensity resampling	0.85	0.82	0.90	0.74	0.78	0.83
Retrained models	PanNET reference model	0.87	0.83	0.90	0.76	0.80	0.84
	PanNET model replicated with I2R	0.87	0.83	0.90	0.76	0.80	0.84
	Alteration 1 — Fat not removed from mask	0.85	0.79	0.85	0.72	0.76	0.80
	Alteration 2 — Intensity resampling formula	0.86	0.78	0.85	0.70	0.75	0.80
	Alteration 3 — Train/Test separate for post processing	0.86	0.81	0.81	0.80	0.81	0.81
	Alteration 4 — Spatial resampling with C3D	0.84	0.72	0.88	0.57	0.68	0.76
	Alteration 5 — Spatial resampling with C3D first	0.83	0.77	0.83	0.70	0.74	0.78
	Alteration 6 — No image windowing	0.79	0.70	0.79	0.61	0.68	0.73
	Alteration 7 — No spatial resampling	0.80	0.73	0.79	0.67	0.72	0.75
	Alteration 8 — Spatial resampling with default interpolator	0.81	0.76	0.77	0.74	0.76	0.76
	Alteration 9 — No intensity resampling	0.81	0.78	0.79	0.76	0.78	0.78

Suppl. Table S2. Overview of the nine altered image processing pipeline configurations evaluated.

Alteration number	Alteration description	Original pipeline	Altered pipeline	Details
1	Removing peripancreatic fat in the segmentation mask	Fat removed	Fat kept	The original model removed peripancreatic fat from the segmentation mask. This alteration omitted that step.
2	Intensity resampling binning functions	PyRadiomics defaults	NumPy library binning functions	Intensity resampling applied using NumPy digitize () function instead of PyRadiomics defaults
3	Testing-set post-processing strategy	Global normalization across all patients in the original model.	Normalization of the testing set performed using statistics from the training set	In the modified pipeline, Z-score normalization in the testing set was performed using statistics from the training set, instead of using global normalization across all patients as in the original model.
4	Spatial resampling implementation	Directly in pyradiomics	C3D (ITK-SNAP)	Spatial resampling was performed using C3D instead of the default resampling method included in PyRadiomics
5	Resampling order in pipeline	At the end of the image processing pipeline	At the beginning of the image processing pipeline	Spatial resampling with C3D was performed at the beginning of the pipeline, before any other preprocessing steps.
6	Removal of image windowing	Soft tissue window (level: 50 HU, width: 500 HU)	No windowing	No intensity windowing step applied prior to radiomics extraction in the altered pipeline
7	Removal of spatial resampling	No	Yes	No voxel spacing normalization applied, images kept at original resolution
8	Interpolation for spatial resampling	B-spline interpolator (default in PyRadiomics)	Linear interpolation	Spatial resampling was performed using linear interpolation instead of the default B-spline interpolator in PyRadiomics.
9	Removal of intensity resampling	No	Yes	No intensity binning applied; full intensity range used directly
